# Supplementary material for: Spatial modelling for population replacement of mosquito vectors at continental scale
Source: PLoS Comput Biol. 2022 Jun 1;18(6):e1009526. doi: 10.1371/journal.pcbi.1009526 (PMC9191746; doi:10.1371/journal.pcbi.1009526)
Supplement: S1 Fig — Time series plot of Site 6 (as in Fig 5) but with a closed population, i.e. no diffusion or advection. The colours correspond to genotype and the line thickness to age class. (PDF) [file pcbi.1009526.s001.pdf]

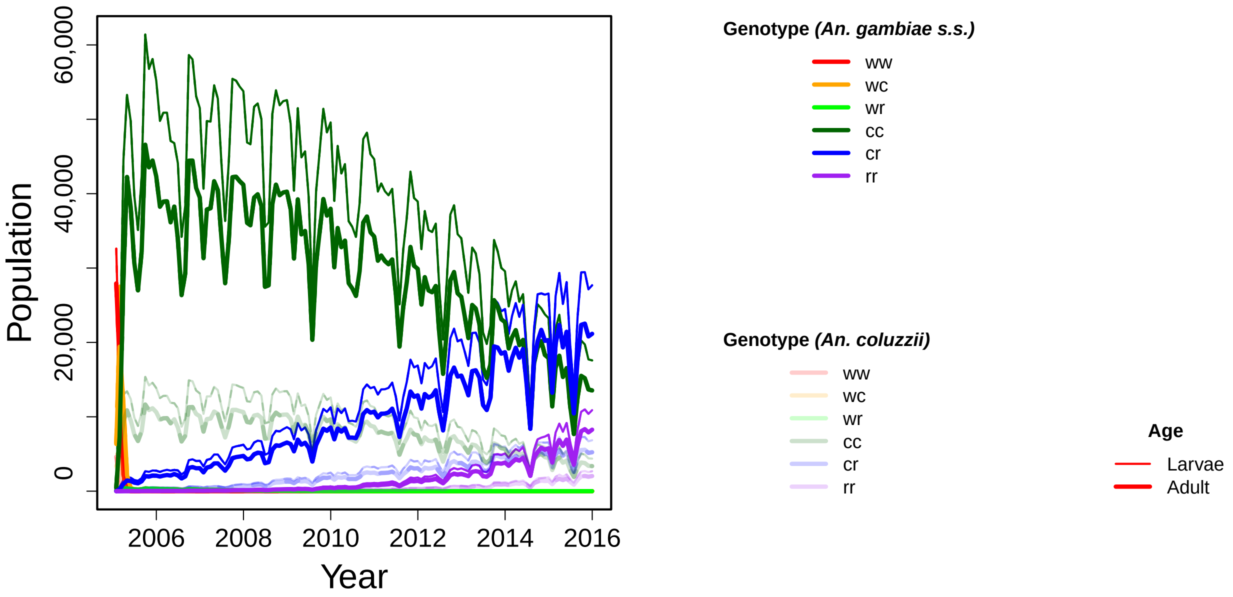

S1 Figure. Time series plot of Site 6 **(as in Figure 5)** but with a closed population, i.e. no diffusion or advection. The colours correspond to genotype and the line thickness to age class.
