# Supplementary material for: Spatial modelling for population replacement of mosquito vectors at continental scale
Source: PLoS Comput Biol. 2022 Jun 1;18(6):e1009526. doi: 10.1371/journal.pcbi.1009526 (PMC9191746; doi:10.1371/journal.pcbi.1009526)
Supplement: S2 Fig — Time series plot of Site 6 as in S1 Fig but with 6 age classes, keeping overall larval emergence period and mortality constant. This result is more closely analogous to a fixed larval emergence time than to the exponentially-distributed version in the main model. The first five age classes combined are plotted here under “larvae”. The colours correspond to genotype and the line thickness to age class. (PDF) [file pcbi.1009526.s002.pdf]

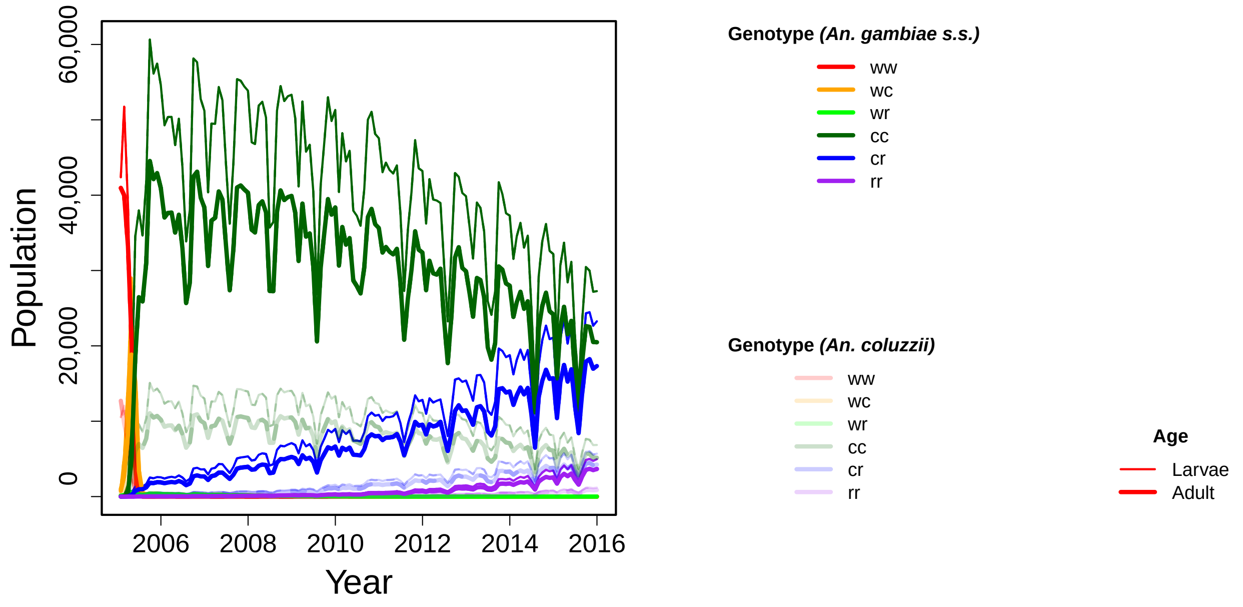

S2 Figure. Time series plot of Site 6 **as in Figure S1** but with 6 age classes, keeping overall larval emergence period and mortality constant. This result is more closely analogous to a fixed larval emergence time than to the exponentially-distributed version in the main model. The first five age classes combined are plotted here under "larvae". The colours correspond to genotype and the line thickness to age class.
