# Supplementary material for: Spatial modelling for population replacement of mosquito vectors at continental scale
Source: PLoS Comput Biol. 2022 Jun 1;18(6):e1009526. doi: 10.1371/journal.pcbi.1009526 (PMC9191746; doi:10.1371/journal.pcbi.1009526)
Supplement: S3 Fig — Time series plot of Site 6 as in S1 Fig but with R(gM, gF) = 0.05 where gM ≠ ww or gF ≠ ww. The colours correspond to genotype and the line thickness to age class. (PDF) [file pcbi.1009526.s003.pdf]

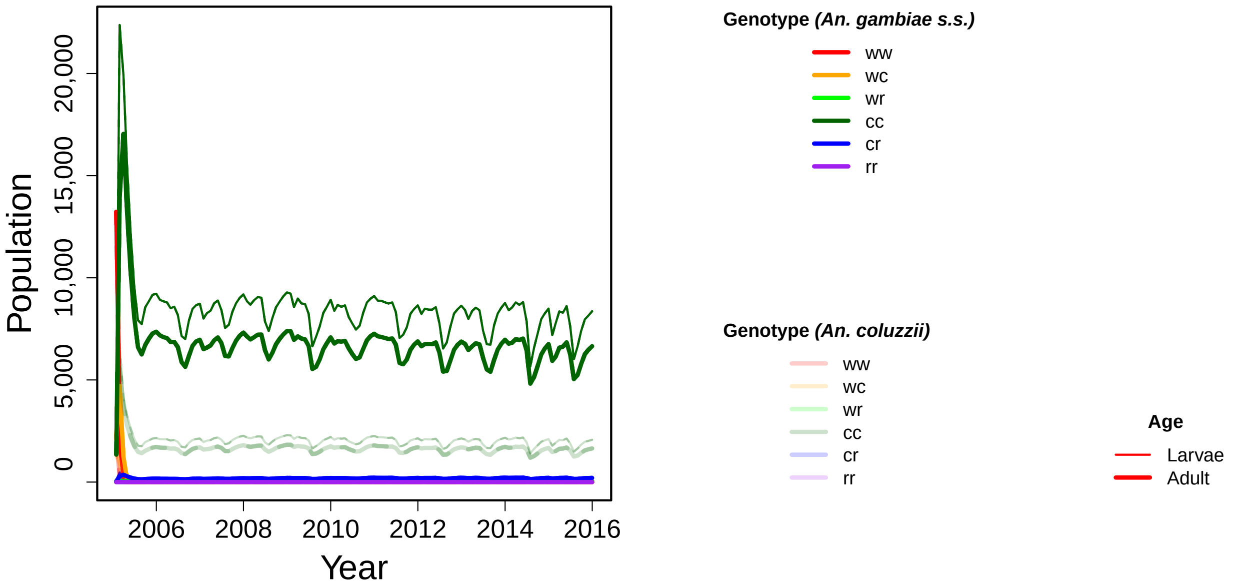

S3 Figure. Time series plot of Site 6 **as in Figure S1** but with $R(g_M, g_F) = 0.05$ where $g_M \neq ww$ or $g_F \neq ww$. The colours correspond to genotype and the line thickness to age class.
