# Supplementary material for: Spatial modelling for population replacement of mosquito vectors at continental scale
Source: PLoS Comput Biol. 2022 Jun 1;18(6):e1009526. doi: 10.1371/journal.pcbi.1009526 (PMC9191746; doi:10.1371/journal.pcbi.1009526)
Supplement: S4 Fig — Time series plot of Site 6 as in S1 Fig but with p(gM, gF, s) modified so that any presence of the genetic construct in the father (wc, cc or cr) results in a 95% male sex bias in offspring. Dashed lines represent males, solid lines females. The colours correspond to genotype and the line thickness to age class. (PDF) [file pcbi.1009526.s004.pdf]

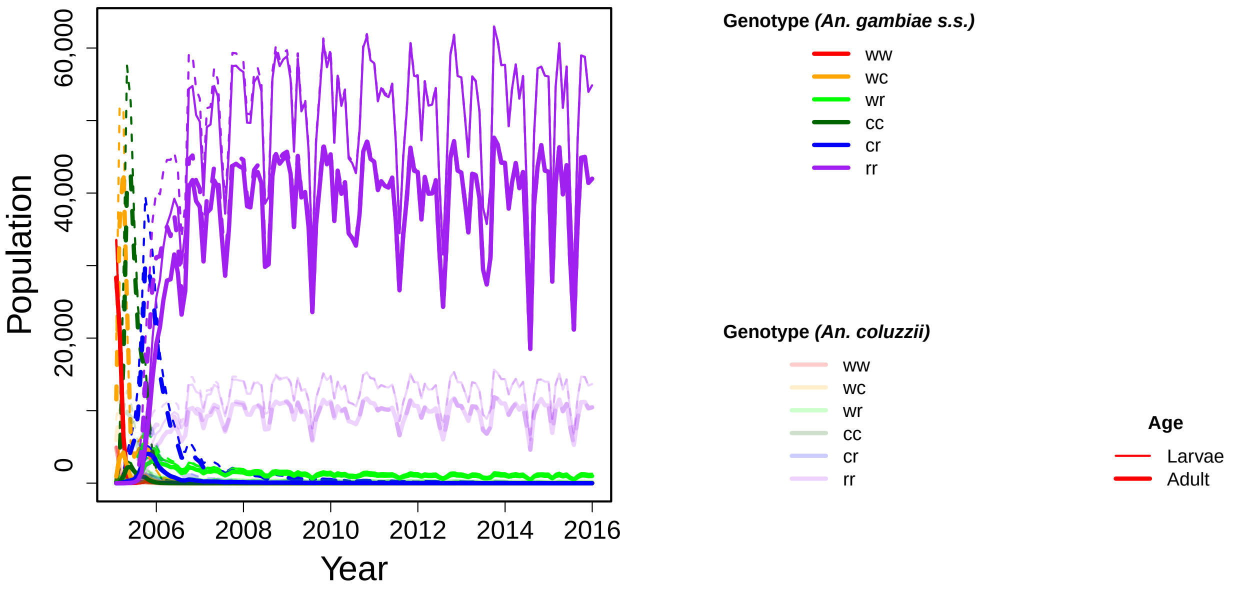

S4 Figure. Time series plot of Site 6 **as in Figure S1** but with $p(g_M, g_F, s)$ modified so that any presence of the genetic construct in the father (wc, cc or cr) results in a 95% male sex bias in offspring. Dashed lines represent males, solid lines females. The colours correspond to genotype and the line thickness to age class.
