# Supplementary material for: Spatial modelling for population replacement of mosquito vectors at continental scale
Source: PLoS Comput Biol. 2022 Jun 1;18(6):e1009526. doi: 10.1371/journal.pcbi.1009526 (PMC9191746; doi:10.1371/journal.pcbi.1009526)
Supplement: S5 Fig — The time series abundance of male mosquitoes at each introduction point as in Fig 5, but using 2 hours instead of 9 hours advection, separated by species, genotype and age (female mosquitoes occur in identical numbers to males in this model). The colours correspond to genotype and the line thickness to age class. (PDF) [file pcbi.1009526.s005.pdf]

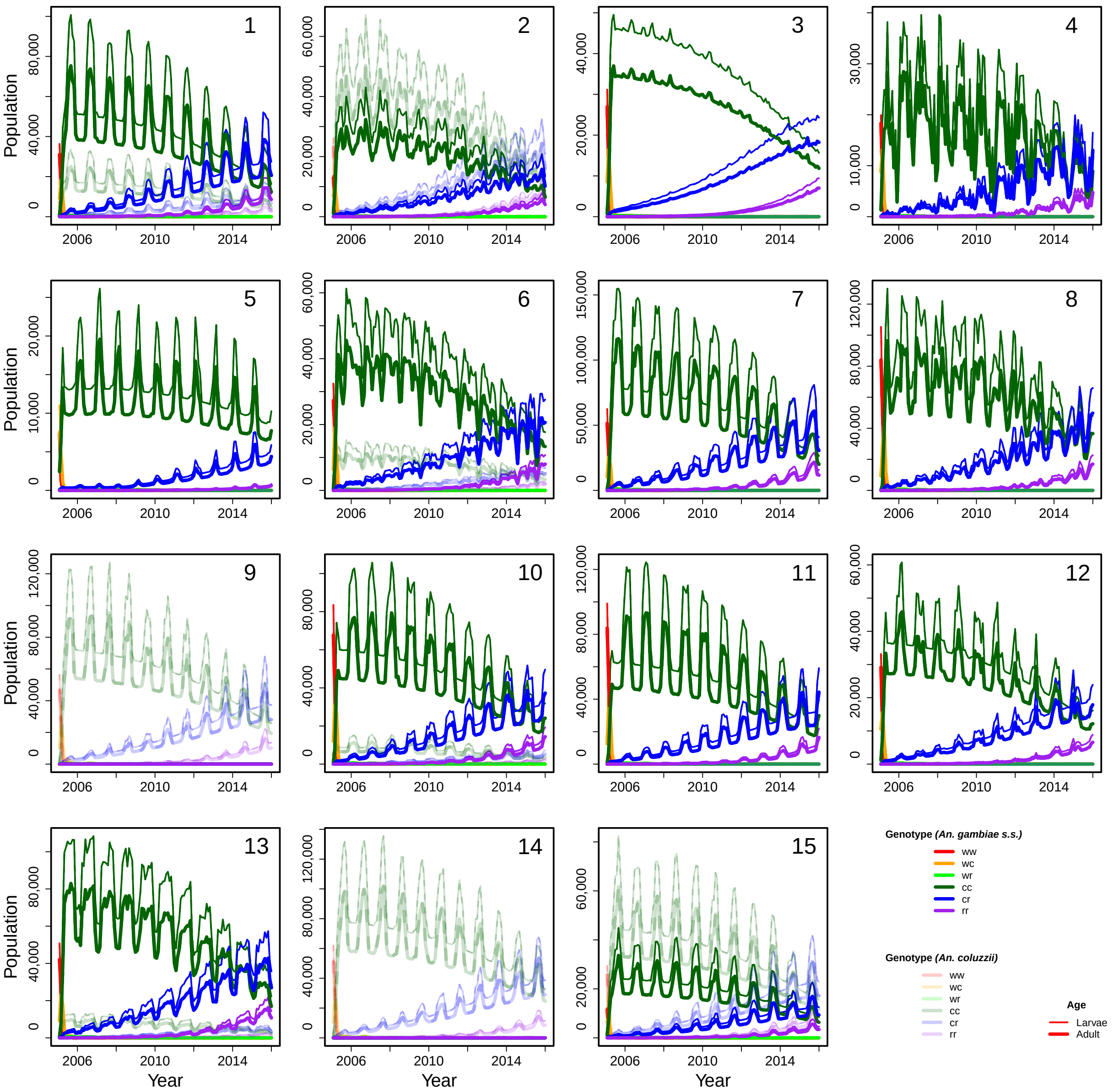

S5 Figure. The time series abundance of male mosquitoes at each introduction point **as in Figure 5, but using 2 hours instead of 9 hours advection**, separated by species, genotype and age (female mosquitoes occur in identical numbers to males in this model). The colours correspond to genotype and the line thickness to age class.
