# Supplementary material for: Spatial modelling for population replacement of mosquito vectors at continental scale
Source: PLoS Comput Biol. 2022 Jun 1;18(6):e1009526. doi: 10.1371/journal.pcbi.1009526 (PMC9191746; doi:10.1371/journal.pcbi.1009526)
Supplement: S6 Fig — The invasion front of the construct at each introduction point as in Fig 4, but using 2 hours instead of 9 hours advection. A separate colour is given for each year. The island introductions are (1) the Bijagós islands (off Guinea-Bissau), (2) Bioko (off Cameroon), (3) Zanzibar (off Tanzania), (4) Comoros (off Mozambique) and (5) Madagascar. Base map from Natural Earth: https://www.naturalearthdata.com/downloads/10m-physical-vectors/10m-coastline/. (PDF) [file pcbi.1009526.s006.pdf]

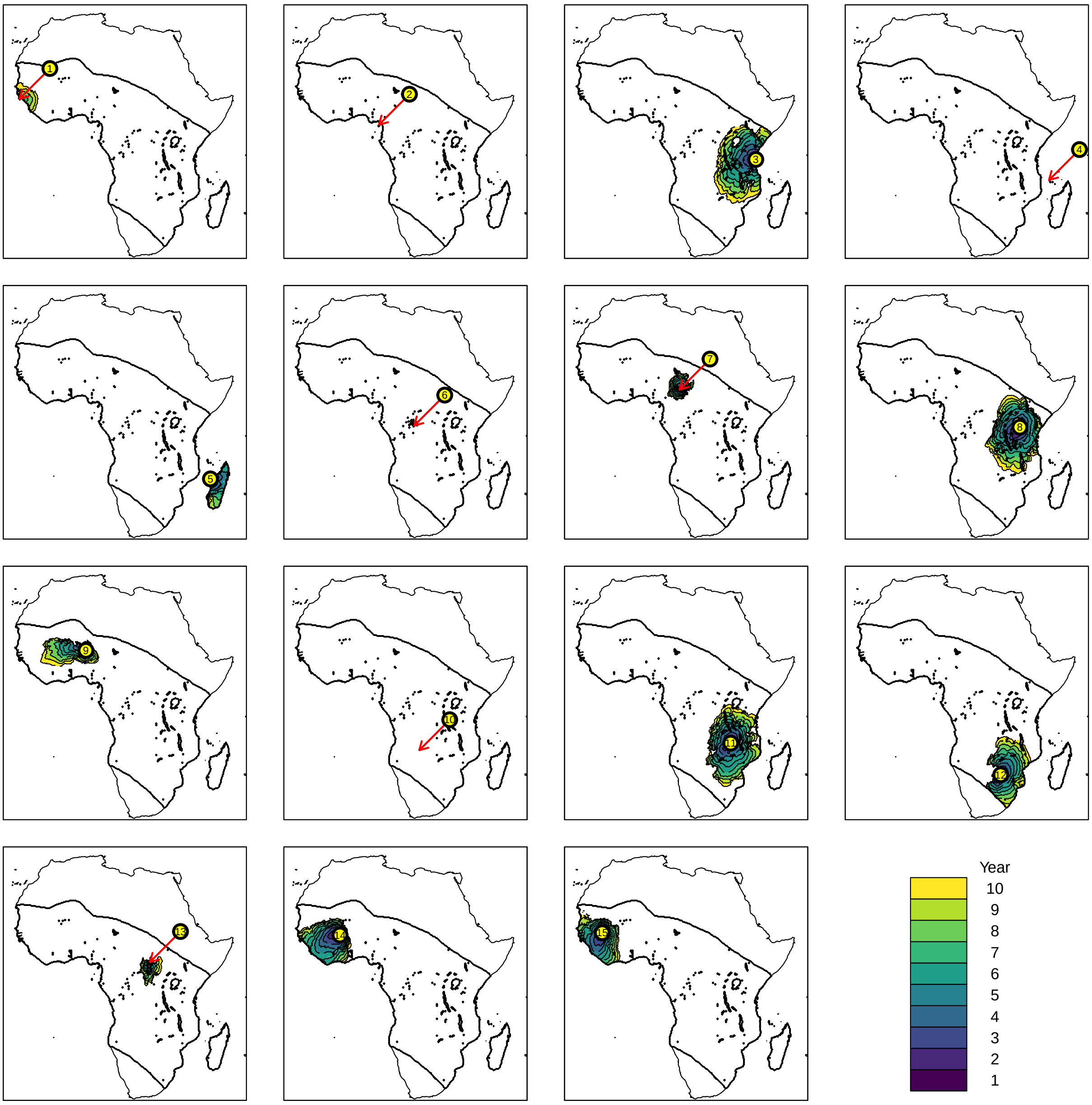

S6 Figure. The invasion front of the construct at each introduction point **as in Figure 4, but using 2 hours instead of 9 hours advection**. A separate colour is given for each year. The island introductions are (1) the Bijagós islands (off Guinea-Bissau), (2) Bioko (off Cameroon), (3) Zanzibar (off Tanzania), (4) Comoros (off Mozambique) and (5) Madagascar. Base map from Natural Earth: `https://www.naturalearthdata.com/downloads/10m-physical-vectors/10m-coastline/`
