# Supplementary material for: Spatial modelling for population replacement of mosquito vectors at continental scale
Source: PLoS Comput Biol. 2022 Jun 1;18(6):e1009526. doi: 10.1371/journal.pcbi.1009526 (PMC9191746; doi:10.1371/journal.pcbi.1009526)
Supplement: S7 Fig — The invasion front of the construct as in Fig 4 for Site 6, but assuming human (and thus mosquito) absence in Sudan, South Sudan and Somalia (national boundaries given in red, as defined by the UN Office for the Coordination of Humanitarian Affairs). Base map from Natural Earth: https://www.naturalearthdata.com/downloads/10m-physical-vectors/10m-coastline/. (PDF) [file pcbi.1009526.s007.pdf]

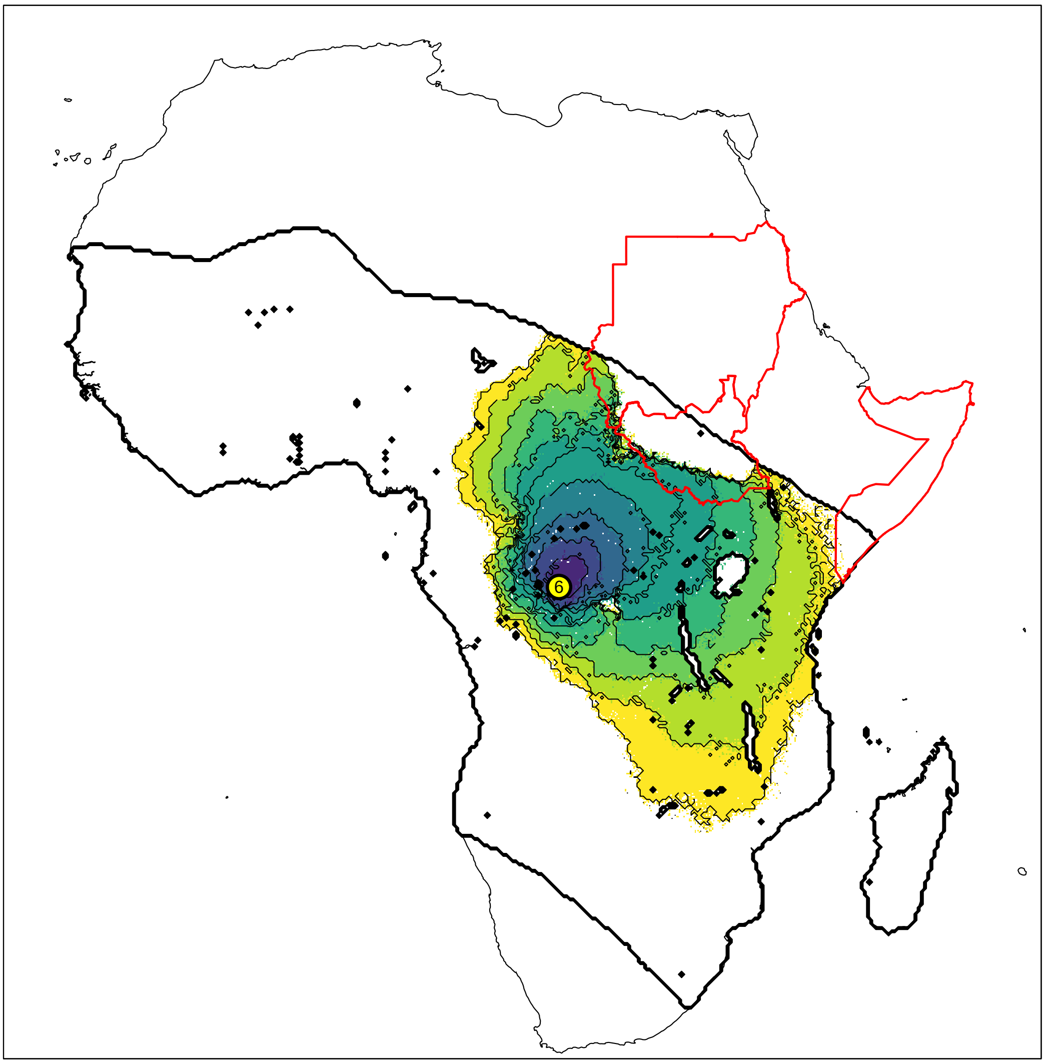

S7 Figure. The invasion front of the construct as in Figure 4 for Site 6, but assuming human (and thus mosquito) absence in Sudan, South Sudan and Somalia (national boundaries given in red, as defined by the UN Office for the Coordination of Humanitarian Affairs). Base map from Natural Earth: `https://www.naturalearthdata.com/downloads/10m-physical-vectors/10m-coastline/`.
