# Supplementary figures and images for: Spatial modelling for population replacement of mosquito vectors at continental scale
Source: PLoS Comput Biol. 2022 Jun 1;18(6):e1009526. doi: 10.1371/journal.pcbi.1009526 (PMC9191746; doi:10.1371/journal.pcbi.1009526)

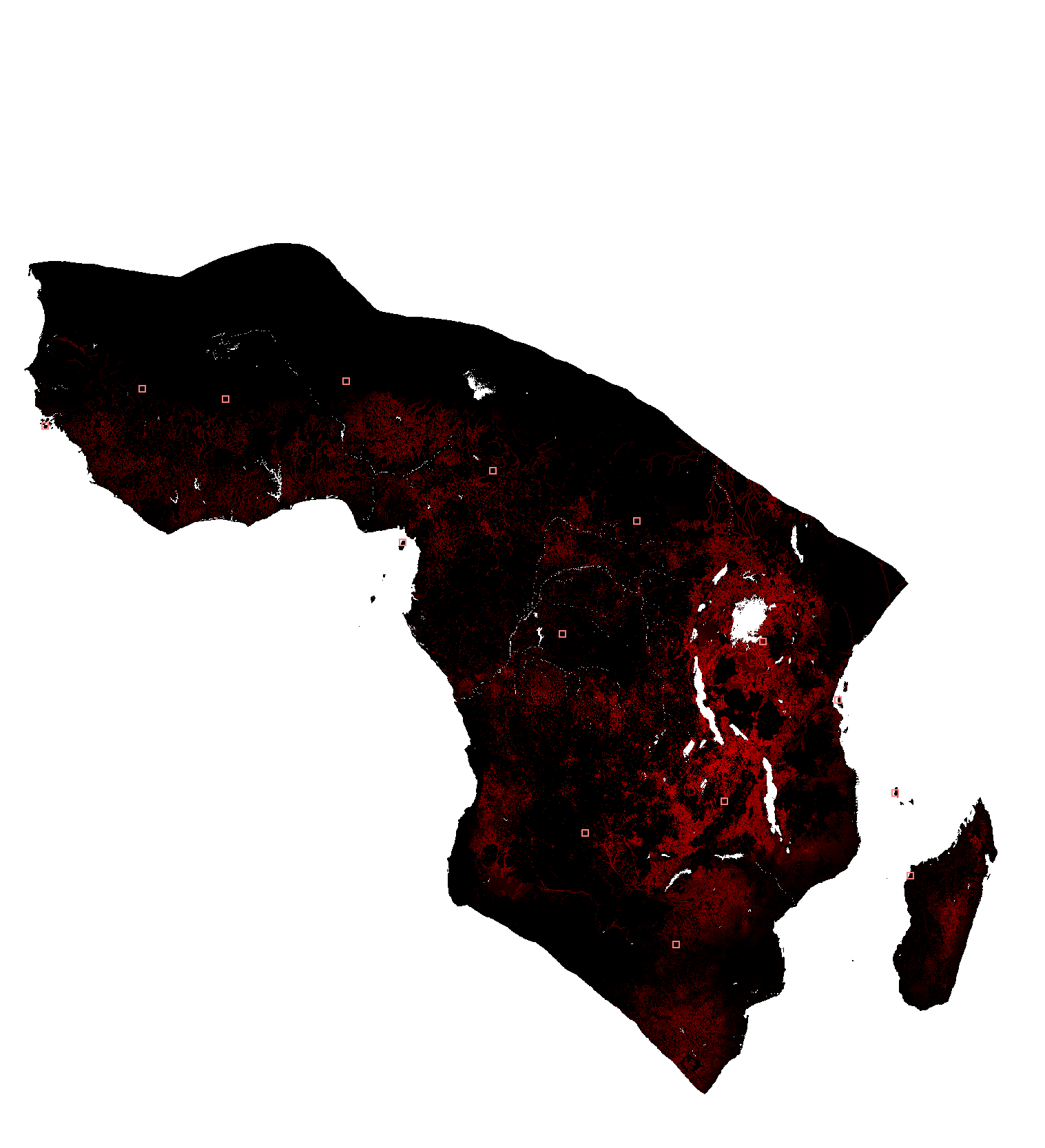

Supplement: S1 Video — (GIF) [file pcbi.1009526.s008.gif]

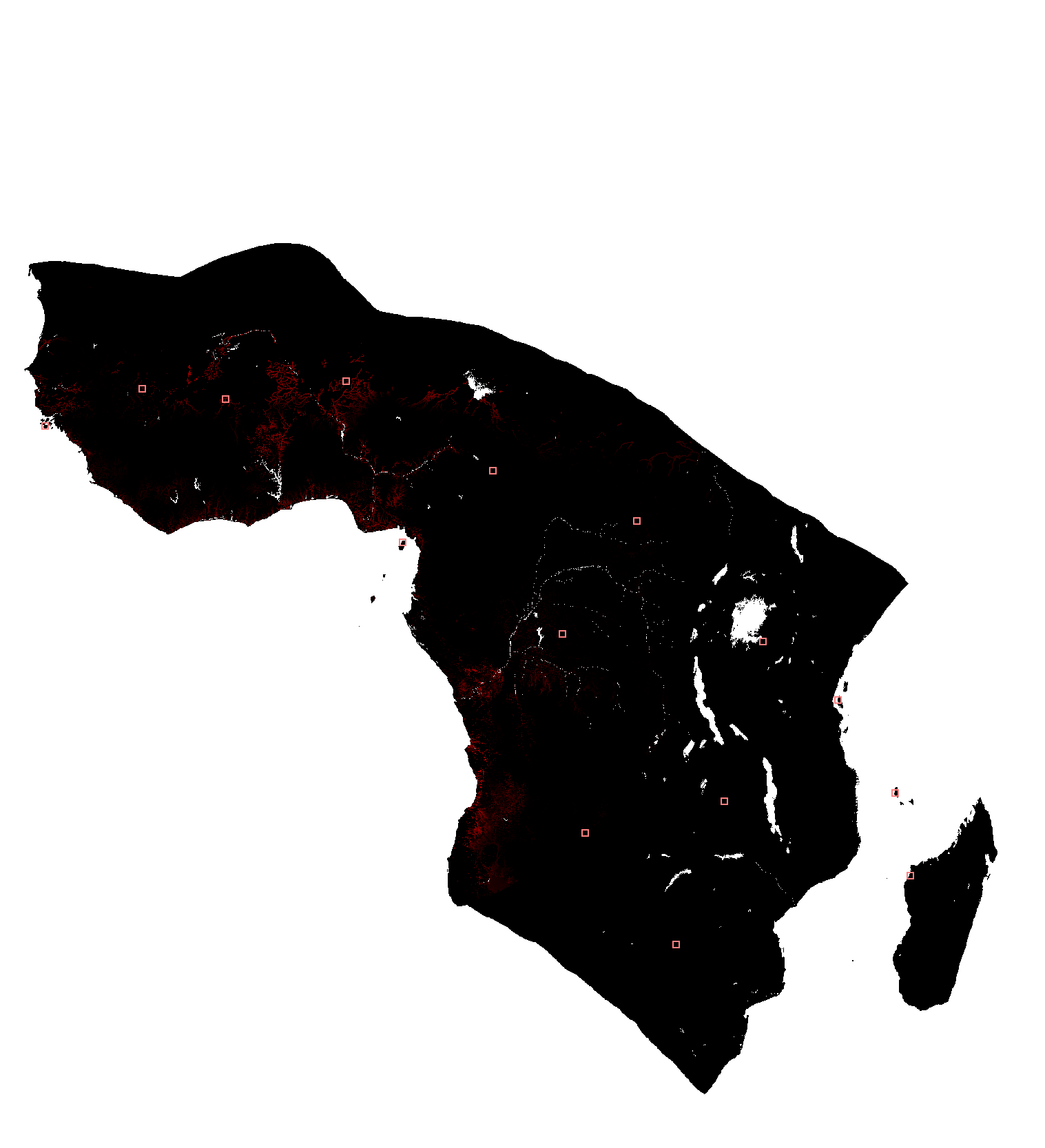

Supplement: S2 Video — (GIF) [file pcbi.1009526.s009.gif]

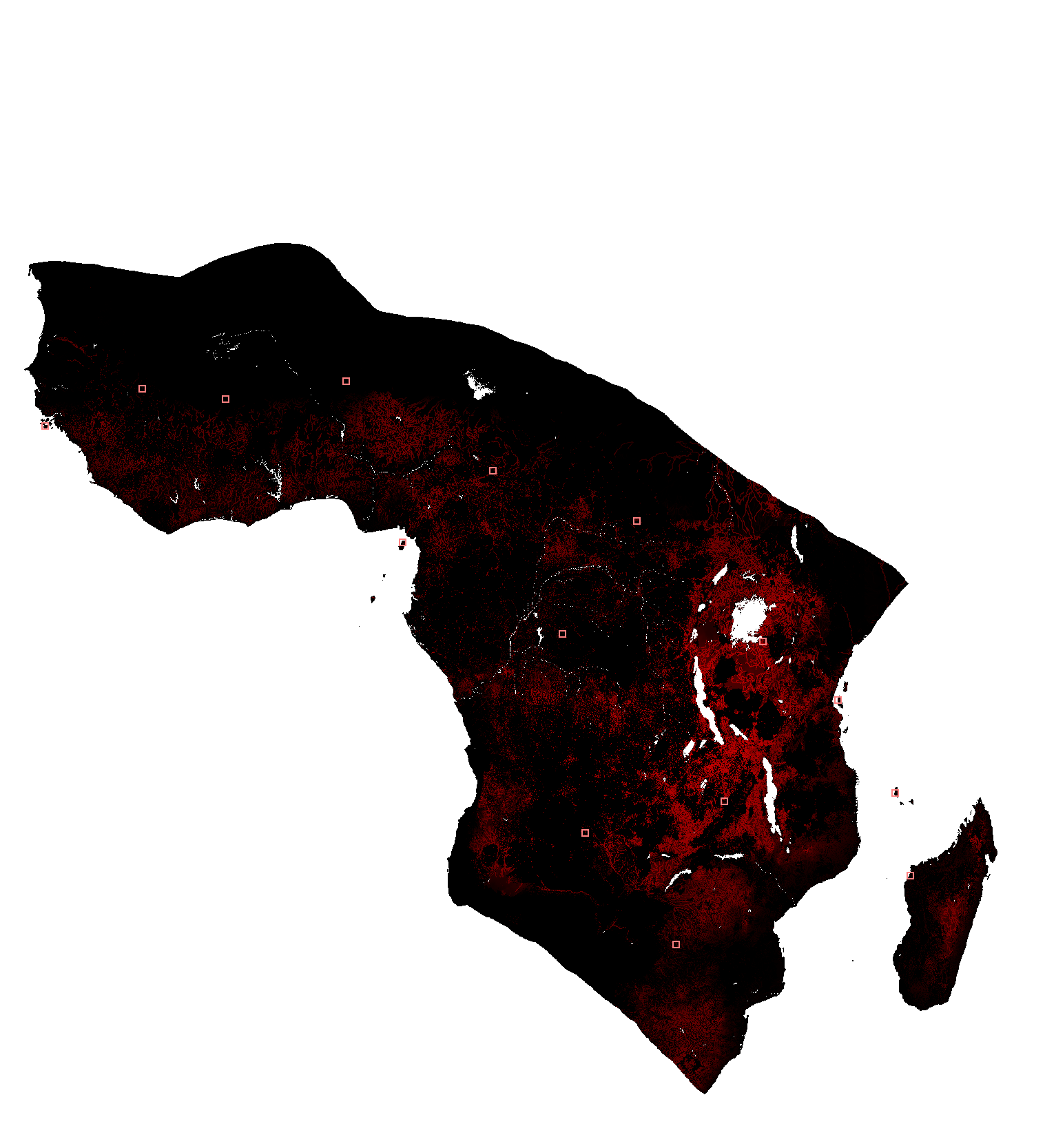

Supplement: S3 Video — (GIF) [file pcbi.1009526.s010.gif]

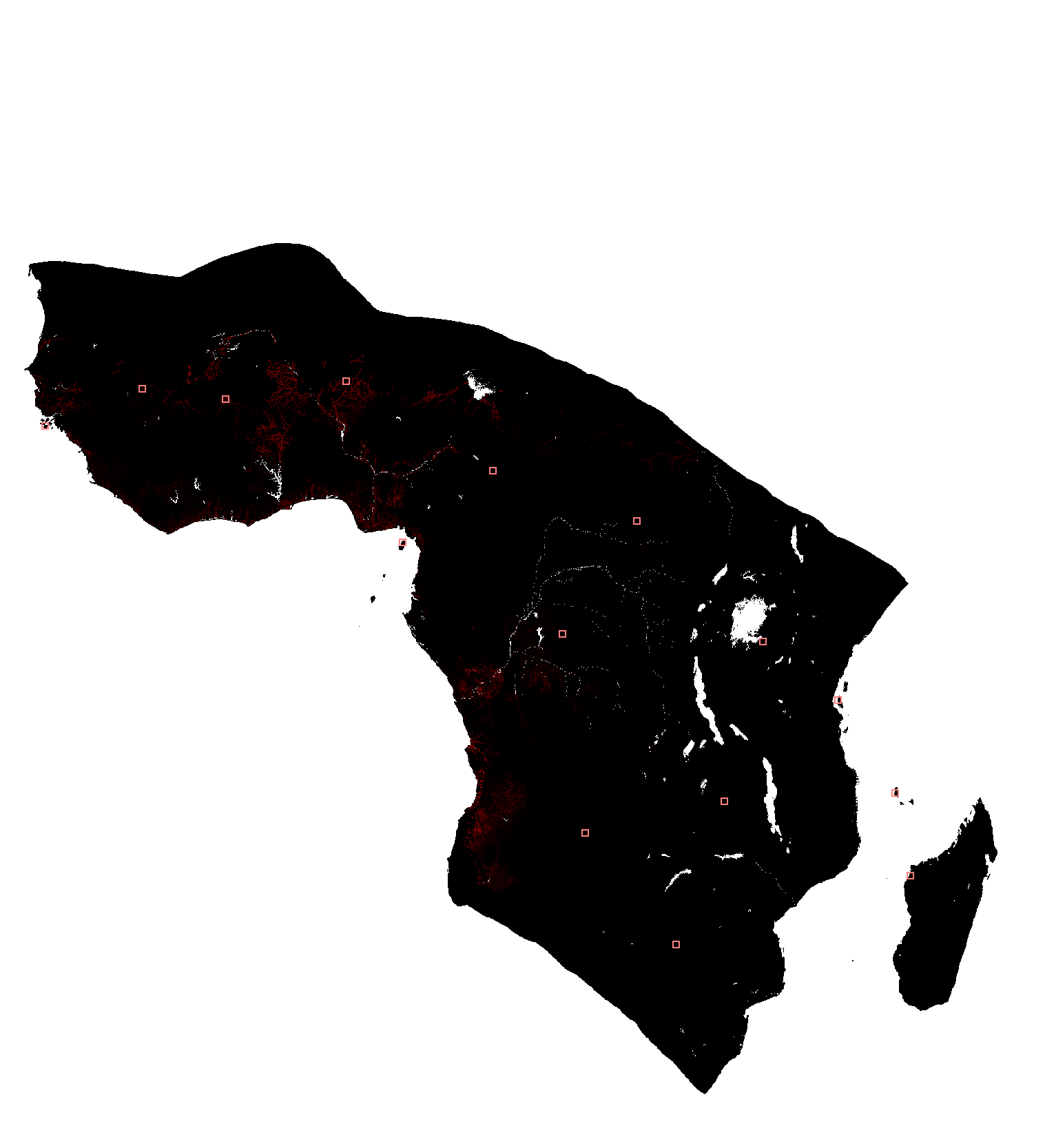

Supplement: S4 Video — (GIF) [file pcbi.1009526.s011.gif]
