# Supplementary material for: Spatial modelling for population replacement of mosquito vectors at continental scale
Source: PLoS Comput Biol. 2022 Jun 1;18(6):e1009526. doi: 10.1371/journal.pcbi.1009526 (PMC9191746; doi:10.1371/journal.pcbi.1009526)
Supplement: S1 Appendix — (PDF) [file pcbi.1009526.s012.pdf]

S1 Appendix: Algorithm for placing introduction sites

As mentioned in the main text, we first select the cells in which at least 10,000 of either subspecies are available year-round on the African mainland. We then randomly select a set Y consisting of 5,000 of those cells as site candidates for ease of computation, discarding the others from consideration. We adapt an algorithm that maximises the probability of animals being captured by a set of traps spread across a domain using gradient descent.

We define a goodness of fit $G(X)$ of a candidate set of 10 release sites X by:

- defining a kernel function $f(d) = \exp(-d/1000)$ where d is distance in km (analogous to probability of detection), then
- for each candidate site $y \in Y$, calculate $p = 1 - \prod_{d(X,y)} (1 - f(d))$ where $d(X, y)$ is the set of distances between each point in X and the candidate y (analogous to the probability of detection by at least one site in X),
- taking $G(X)$ as the sum of p over all the candidate sites (analogous to the expected number of candidate cells in Y detected by the sites in X).

For 20 iterations, we:

- initially select X to be 10 sites selected at random from Y, and
- calculate $G(X)$ for this initial X.
- For each of the currently selected release sites $x \in X$:
  - pick the 20 spatially closest candidates to x and recalculate $G(X)$ for each, temporarily replacing x with the nearby candidate in the set X; and
  - permanently replace x with the candidate that gives the highest $G(X)$ in the set X, if higher than the current value.
- If the set X has changed after going through all of the release sites, then repeat the process.
- Otherwise, stop, outputting the final set X and final goodness of fit $G(X)$.

Of the 20 output sets X, we then use the set that gave the highest value for $G(X)$.
