# Supplementary material for: Spatial modelling for population replacement of mosquito vectors at continental scale
Source: PLoS Comput Biol. 2022 Jun 1;18(6):e1009526. doi: 10.1371/journal.pcbi.1009526 (PMC9191746; doi:10.1371/journal.pcbi.1009526)
Supplement: S2 Appendix — (PDF) [file pcbi.1009526.s013.pdf]

S2 Appendix: Neural network details

The inputs of the neural network were normalised by the mean and standard deviation of each predictor. The network was given three hidden layers of size 40, 20 and 10 (arrived at by experimentation; these sizes gave near-optimal validated errors: see Table 3 in text); a rectified linear (ReLu) activation function for the hidden layers, and a sigmoid activation function for the output neuron to produce a probability of the predictors producing a mosquito of *An. gambiae* s.s. as opposed to *An. coluzzii*. We use a cross-entropy loss function, such that the neural network effectively finds the maximum likelihood of the neuron weights given the observed subspecies at each site. The neural network is implemented using the Keras package [1] in the R programming language [2]. We use a 75:25 training-testing split on the data, using the DUPLEX algorithm [3] to ensure a spread of geographic locations for both training and testing sets. We then run the neural network for 200 epochs, and use the minimum validated loss to determine the number of epochs to subsequently run the model for.

For each tested set of predictors in the forward selection process, we train the neural network 500 times, running for 200 epochs each time. We calculate the validation loss after each epoch, take the minimum as our measure of goodness-of-fit for that trained network, and take the mean of the 500 validation losses obtained in this way. This is to account for variations in neural network effectiveness caused by the stochastic gradient descent process. Starting with an empty set of predictors, the predictor whose inclusion gives the best mean validation loss is accepted, until accepting new predictors no longer reliably decreases the validation loss.

We then train a neural network on the final selected set of predictors 100 times, taking the pointwise mean of the 100 runs as our final estimate of relative abundance at each location. This model averaging is performed in order to ensure that our final ensemble estimate is representative of the neural network modelling process as a whole: as the optimisation process is stochastic, a single network may differ substantially from another, especially where little data is available.

References

1. Chollet, F., Allaire, J., et al. (2017). R Interface to Keras. https://github.com/rstudio/keras.
2. R Core Team (2020). *R: A Language and Environment for Statistical Computing*. R Foundation for Statistical Computing,Vienna, Austria.
3. Snee, R. (1977). Validation of regression models: Methods and examples. *Technometrics*.
