# Supplementary material for: Spatial modelling for population replacement of mosquito vectors at continental scale
Source: PLoS Comput Biol. 2022 Jun 1;18(6):e1009526. doi: 10.1371/journal.pcbi.1009526 (PMC9191746; doi:10.1371/journal.pcbi.1009526)
Supplement: S3 Appendix — (PDF) [file pcbi.1009526.s014.pdf]

S3 Appendix: Description of illustrative animations

GIF animations of the simultaneous introduction of the construct at all sites are available in Supporting Information Files: `S1_Video.gif`, `S2_Video.gif`, `S3_Video.gif`, `S4_Video.gif` show the animations for 9 hour wind advection for *Anopheles gambiae* s.s. and *Anopheles coluzzii*, and the 2 hour equivalents for both subspecies, respectively. The release points are marked as pink squares. Cells are colour coded: the amount of red in a cell represents the relative number of mosquitoes of the given subspecies with a wildtype w allele. Green and blue similarly represent construct c and resistance r respectively.
