# Supplementary material for: Spatial modelling for population replacement of mosquito vectors at continental scale
Source: PLoS Comput Biol. 2022 Jun 1;18(6):e1009526. doi: 10.1371/journal.pcbi.1009526 (PMC9191746; doi:10.1371/journal.pcbi.1009526)
Supplement: Attachment — Submitted filename: response to reviewers final.docx [file pcbi.1009526.s015.docx]

Thank you for the editor and reviewers for your kind words and useful feedback regarding our manuscript. It represents a lot of work from all of us, so it was gratifying for us to see that it resonated with all of you.

The relevant parts of the reviewers’ responses are below, followed by our responses.

**Reviewer #1**

**The model description was generally clear, though some parts could have been even clearer (details below).**

**This in itself is an ambitious task and it wasn’t entirely clear to me why the authors chose to do it rather than use the output of Tene Fossog et al (20** **$K_1(t, \mathbf{x})$ and $K_2(t, \mathbf{x})$15), that they cite as a comparator (more on this below).**

We have added text that reiterates our reasoning for adapting the Tene Fossog et al. (2015) output – basically we wanted to make it more robust to being applied to a continental scale, which it wasn’t in the original paper.

**In addition the numerous demographic parameters are set from various sources (many of which are highly uncertain).**

We fully agree. The model provides a platform for further sensitivity analysis (by ourselves or others) and also represents a stepping stone for us towards a fully Bayesian assessment, to account for such uncertainties. We have made some references to this and to the prospect of sensitivity analyses in the paper.

**I think the paper is more successful if the first aim is the principle one and I’d suggest rewording to place greater emphasis on it – describing a model that can be applied to other questions will no doubt be valuable to ongoing discussions about mosquito gene drives. The specific results that are presented can serve as an example of the use of the model.**

Yes, the first aim is definitely our principal target. We have added some text in the Abstract, just before Section 1.1, and in the first paragraph of the Discussion to make this clearer.

1. **Could you give more detail of how the diffusion aspect of the model works on the lattice – what is the numerical procedure? An illustrative diagram of this (and the model mechanics more generally) would be very helpful.**

We have added a subsection Section 2.1.4 to briefly describe the diffusion process – as it is a very common approach to modelling diffusion, we have not provided an illustrative diagram specifically for this as these are widely available in the literature.

2. **Relatedly, it wasn’t clear to me how the carrying capacity of a cell is modified by the presence/absence of human populations? Is it zero if there is no settlement and? And is there a difference if there is one or two or more settlements?**

Yes, it is assumed to be zero if there are no human settlements at all within a 5 km x 5 km grid cell but not once a grid cell is assumed to be habitable by mosquitoes (by virtue of some human presence). Carrying capacity is governed by precipitation and the availability of permanent and intermittent water bodies rather than the density of human settlements. We have added several sentences in Section 2.2.1 to clarify this.

3. **The relative abundance methods section was difficult to follow. Maybe you could start by stating the entomological data, and the environmental predictors you will apply? I was quite lost in the second paragraph “Using our version of the predictors…”, and I suspect others will be unless they are very familiar with these methods. I’d suggest replacing this section with a biology-focussed summary of what you did, and an appendix that spells out the details.**

We have amended the text for clarity and now describe the entomological data used in Tene Fossog’s paper, list the environmental predictors they used, and clarify “Using our independently sourced version of the predictors”. Again, we also note that we sourced the same predictors independently to allow us to apply our analysis at a larger spatial scale (all of sub-Saharan Africa). We have also moved much of the technical detail regarding the neural networks to an appendix.

4. **Similarly, the relative abundance results section is very technical and focusses on the statistics (details that could be in an appendix) rather than the biology. Fig 1 is not very clear and I’d suggest revising and moving to appendix. What I was hoping for was a figure showing how the results differ from those of Tene Fossog et al – could you re-work fig.2 to to show this? Comparison of the two models was a stated aim of the exercise, but it was not very present in the results or discussion.**

We have now revised Figure 1 and reworked Figure 2 to include Tene Fossog et al.’s results for direct comparison.

**Minor

1. Pg 6: “persistence of transgenic mosquitoes from release sites”.**

Fixed.

**2. Pg. 7: “ both spatially and temporally varying carrying capacities driven by precipitation and land use factors” is described as a “hypothesis”. I would describe it as a statement (a fairly obvious one at that). This paragraph needs re-wording.**

Excellent point – we were referring to the way in which the literature had characterised the behaviour of these carrying capacities, but did not make this clear. We have replaced “both” with “the nature of” to clarify this.

**3. Throughout ms – why do you refer to An. gambiae and An. coluzzii as “subspecies”, they are commonly agreed to be different “species”?**

The reviewer is correct, though our reference is an intentional one to emphasise the lack of complete reproductive isolation between them, and the term “species” is associated with multiple competing definitions. We now explain our reasoning for the use of the term in the text.

**4. Pg. 12. transforming <1 mosquito → 0 mosquitoes is not “approximating stochastic effects” – it is a deterministic rule that allows extinction to occur in the model.**

Fixed.

**5. Table 1 misses out a lot of the model parameters – e.g. construct parameters and K parameters. Would be nice to have them all in 1 table, possibly with subsections.**

This is an excellent point - we have done this as far as we believe is practical, by including the parameters taken from Beighton et al. (2017) and North and Godfray (2018), and adding subsections to the table. We believe describing the other parameters in a table, as they are not explicit numbers, would be distracting and are better done in text.

**6. Eqn 2.4 - Why don't you write J as a function of mM,mF,gM ?**

We avoided using the parental classes in the function definitions to avoid the notation becoming overwhelming. We have now added a sentence after Equation 2.3 to explain this more explicitly.

**7. Eqn 2.7. Can you explain the different fitness parameters : he,hn,se,sn. Why is the heterozygote cost quadratic?**

These are now described in Table 1 with the other parameters. Though we had originally modelled the heterozygote cost as quadratic (because of the separate effects of the nuclease and effector in the gene drive described in Beaghton et al. (2017)), it is not quadratic in our case as we are modelling a nuclease but not an effector gene in our model. We have now changed the parameter values and rerun the simulations, and correspondingly changed the results and discussions where applicable. Thank you for picking up this important oversight.

**8. Eqn. 2.9. alpha0 is a term for permanent larval habitat, not for aestivation.**

Thank you, well spotted. This has now been fixed.

**9. how are the relative abundances implemented in the model equations? Is it via the alpha_ij terms?**

No – by combining our results from the absolute and relative abundance subsections (2.2.1 and 2.2.2). We have added some text in the latter section to make this clearer.

**Reviewer #2:**

**- Section 1.1 describes the gene drive system as 'neutral' and 'fitness-neutral, but it seems from 2.7 that there are fitness costs. This contradiction should be rectified. ('neutral' is also used in the discussion).**

Excellent point – we follow the perspective of Beaghton et al. (2017) and Hammond et al. (2016) that there will be some unavoidable fitness costs in the reproductive process when introducing a gene drive construct into a mosquito population. We have now clarified our language throughout, see Section 1.1 for a summary of the construct and fitness assumptions. **- The model is described as a PDE model with 1 day time steps. For the non-mathematician, it would be good to add a sentence for the reader how this differs from a difference equation model (if it does).**

It does differ in that we use a Runge-Kutta scheme instead of a forward Euler approach. A sentence has been added to clarify this, and the numerical suitability of the timestep.
 **- In the results it is said that when addition is for 2 hours, only Zanzibar introductions reached the mainland, whereas in the discussion it is said that Bioko introductions also reach the mainland. This contradiction should be rectified.**

Thank you for noticing this oversight. We unintentionally omitted an illustrative Supplementary Material Figure which has now been added, and the contradiction has been rectified. **- The model has only 3 alleles, which is fair enough, but perhaps it could be mentioned in the discussion that Beaghton et al. (2017) found that if one allowed mutations to disrupt the effector while leaving the driver intact, then eventually (far from the release site) one ends up with effector-less drivers, and in this sense the results presented in this paper may be optimistic. Any further thoughts on how the model might be extended would also be welcome.**

We agree that the results in the paper may be optimistic for a variety of reasons (including for example the absence of population structure, allelic variation and at the drive’s target locus, and hence the existence of native resistance to the drive). This choice is deliberate to provide an “upper bound” or “optimistic” prediction for a population replacement gene drive system given an idealised effector with negligible fitness cost. We have clarified this intent in the introduction. We have also added emphasis that the main purpose of the model is to provide a platform for further sensitivity analysis. Without explicitly including the effect of mutations to the effector component into our model, however, we cannot be sure to what effect these mutations will have on the overall dynamics because (inter alia) we do not know the effect of the subsequent change in the fitness cost of effector expression.
