# Supplementary material for: Spatial modelling for population replacement of mosquito vectors at continental scale
Source: PLoS Comput Biol. 2022 Jun 1;18(6):e1009526. doi: 10.1371/journal.pcbi.1009526 (PMC9191746; doi:10.1371/journal.pcbi.1009526)
Supplement: Attachment — Submitted filename: response to reviewers part 2.docx [file pcbi.1009526.s016.docx]

• Figure 1 is slightly clearer than before but still fairly confusing. It needs a more intuitive description of how to interpret and what it is telling us – starting with a more intuitive y-axis. ( To the uninitiated, it is not clear whether loss is good or bad..). The legend is inaccurate – there are no small dots/ labelled dots.

We have tweaked Figure 1 to make it clearer, and added more information for interpretation in the figure caption.

• Which of the 4 distance metrics (page 22/23) are the final results based on? all of them? In which case how are they combined?

The neural network is trained using cross-entropy as its goodness of fit. This was briefly mentioned in the description of Table 1 in Section 3.1.1, but is now also mentioned in the Methods in Section 2.2.2 and in the description of the measures themselves earlier in Section 3.1.1.

• Figure 2. your map does not exclude areas where there are no gambaie or coluzzii mosquitoes, giving the impression that colluzzii is dominant in - South Africa, Madagascar, Spain (?). How do you account for this? Contrast with Fig 3 where you have species range boundaries, and blank outside them.

The map in Figure 2 describes relative abundance. The species range boundaries are estimates given by the Malaria Atlas Project and thus may not be 100% accurate, so giving more complete data of the modelled relative abundance may still be useful outside of those boundaries. In addition, the results of Tene Fossog et al. (2015) are also outside of the boundaries, and should be reported in full. A sentence has been added to the figure caption: “Note that the relative abundance estimates cover areas where neither subspecies are expected to exist.”

• Also Figure 2, I’m not sure how to interpret (c) - The standard deviation of relative abundance. Can you explain this in a more intuitive way – is it a measure of uncertainty in the prediction, or predicted variation in the presence of each species? The legend could be clearer on this.

It does measure uncertainty in the prediction – the caption has been modified to specify this.

• Will you make the predicted layer for relative abundance freely available to other researchers? This would be useful.

Yes – all data and code will be available in the final published version.


Minor comments/ suggestions

• In the author summary the wording “ ..eventually stopping due to the emergence of resistant alleles.” struck me as quite clumsy and not a good description of what happens. “the drive allele is eventually ousted by a resistant allele” or some such would be more accurate.

This has been fixed, along with some other wording changes.

• Page 3 ‘North & Godfray 2018’ does not model a suppression drive – I think its the wrong reference (North et al 2020?).

Thank you, well spotted. Fixed.

• Page 7 “ information has been generally used” what does ‘generally’ mean here?

We have replaced this term with the less ambiguous “widely”.

• Page 9. Surely m=N_subspecies -1 (you don’t have the -1)?

Well spotted again! Fixed.

• Also p9. “the constant transition rate b results in an exponential distribution of maturation between larval classes”. More accurate to say here “maturation from larvae to adult”.

This was meant to say N_age >= 2, which is now fixed, and your point incorporated.

Reviewer #2: Responded very well to the previous comments. No further comments

Thank you for your previous helpful review, and glad we could address your comments fully!
